# Supplementary material for: On neighbourhood degree sequences of complex networks
Source: Sci Rep. 2019 Jun 6;9:8340. doi: 10.1038/s41598-019-44907-8 (PMC6554413; doi:10.1038/s41598-019-44907-8)
Supplement: Supplementary file 1 — Supplementary Material [file 41598_2019_44907_MOESM1_ESM.pdf]

Supplementary material for "On neighbourhood degree sequences of complex networks"

Keith M. Smith[1,*]

[1]Usher Institute of Population Health Science and Informatics, University of Edinburgh, 9 BioQuarter, Little France, Edinburgh, EH16 4UX, UK
[*]k.smith@ed.ac.uk

ABSTRACT

The following supplementary material provides the analysis of the novel neighbourhood degree sequence indices on random graphs, random geometric graphs, small-world and scale free networks and random hierarchy models.

1 Models

For each of the models below, networks with sizes of 16, 32, 64, 129 and 256 were generated. Each model was generated a number of times and the average index value for each network size and parameter value (if relevant) was computed.

Random graphs: Erdös-Rényi random graphs[1] were generated by constructing adjacency matrices with uniformly random values in $[0,1]$. Fifty of each size were generated and these were binarised at 20% density.

Random geometric graphs: Random geometric graphs[2] were generated by choosing x and y co-ordinates uniformly at random in $[0,1]$ and computing inverse distances between them to get weighted adjacency matrices, $w_{ij} = \exp(-d_{ij}^2/4)$. Fifty of each size were generated and these were binarised at 20% density.

Small-world models: Watts-Strogatz small-world models[3] were generated for different levels of randomisation– 10% to 90% in steps of 10%. For each network size and level of randomisation, 25 realisations of the model were generated.

Scale-free models: Albért-Barabasi small-world models[4] were generated for core sizes of $n/8$, $n/4$, $3n/8$ and $n/2$. For each network size and core size, 25 realisations of the model were generated.

Random hierarchy models: Random hierarchy models[5] were generated with 3 hierarchical levels and hierarchy strength parameters ranging from 0.1 in steps of 0.1 up to 0.9. For each network size and parameter value, 25 realisations of the model were generated.

2 Results

Values of neighbourhood similarity, relative node heterogeneity, neighbourhood organisation and both versions of hierarchical complexity were computed for small-world, scale-free and random hierarchy models and presented in Fig 1. We shall comment on each index in turn.

Neighbourhood similarity showed a general trend in these models of being very small for larger network sizes, indicating that neighbourhood similarity in large graphs is a rare property, Fig 1, column 1. We observed that small-world neighbourhood similarity is larger for less randomised graphs. This makes sense since low values of randomisation in small-world models provides graphs more similar to the highly symmetric regular lattice on which the model is based. Similarly, random hierarchy neighbourhood similarity was large for higher hierarchy strengths, which reflects the fact that large values of hierarchy strength provides a highly ordered hierarchical structure where edge weights between and within hierarchy levels become linearly separable[6]. Both small-world and scale-free models were highly asymmetric for network sizes of 64 and above, whereas random hierarchy models held on slightly more to symmetric properties with increasing n.

Relative node heterogeneity of small world models remained around 1 for all randomisation values, indicating that small-world degree distributions display a fractal property with local neighbourhood degree distributions resembling the degree distribution of the entire network, Fig 1, column 2. Random hierarchy models displayed moderate values of relative node heterogeneity, with higher hierarchy strength providing node heterogeneity at less than half the value of global network heterogeneity and lower hierarchy strength displaying a similar fractal-like node heterogeneity as for small-world models. Scale-free models on the other hand, tended to have higher node heterogeneity than global heterogeneity. This was up to twice as high for lower core sizes.

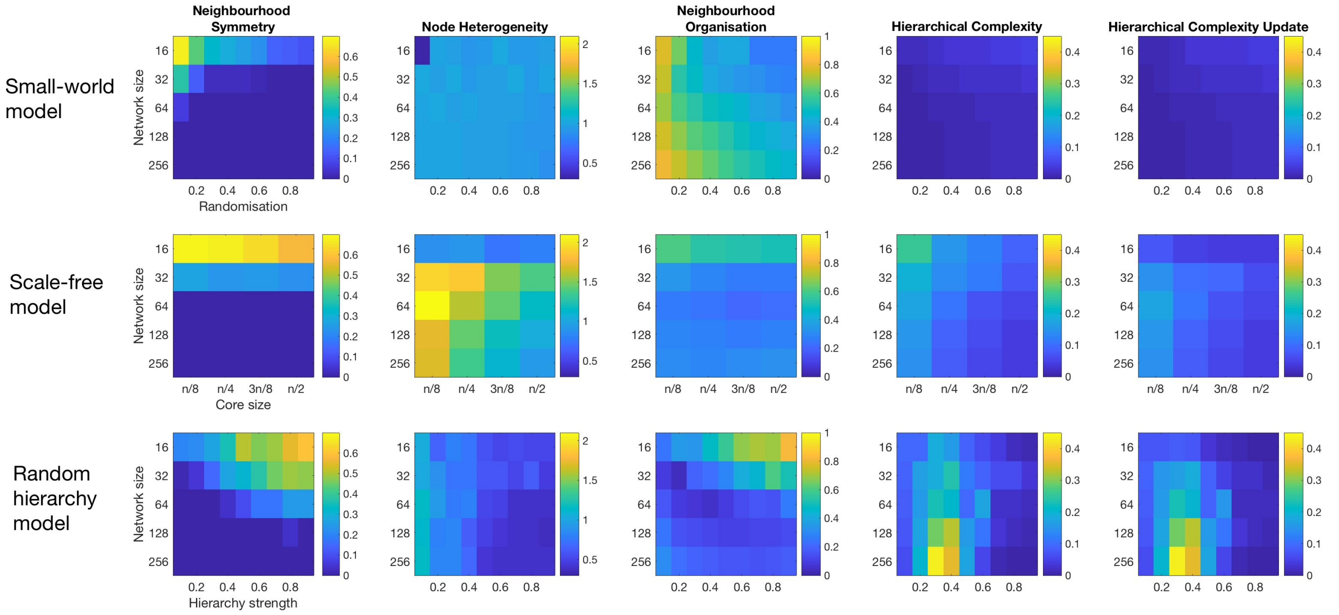

Figure 1. Index values of neighbourhood degree sequence indices (indicated by column titles) for small-world, scale-free and random hierarchy models (indicates by row titles) for different network sizes (y-axis) and model parameters (x-axis).

Neighbourhood organisation appeared to show some similar traits to neighbourhood similarity except that measurable values were still obtained for large networks, Fig 1, column 3. For example, just looking at network sizes of 16 and 32 showed very similar patterns of neighbourhood organisation in these models as for neighbourhood similarity. The notable exception was that size 16 scale-free models showed a very large amount of neighbourhood similarity and yet a much more moderate amount of neighbourhood organisation, relatively. On the other hand values of organisation reaching above 0.4 were still prevalent at network sizes of 128 and 256 for these models. This implied that although organisation contains within it some of the principles of symmetry, and indeed symmetric graphs are organised, organisation is also very possible in graphs with highly asymmetric neighbourhoods.

The updated hierarchical complexity, R_{Ω}, did not show drastic differences to the original R in these models, Fig 1, columns 5 and 4, respectively. One notable difference in smaller networks of size 16 was a decrease of values once neighbourhood organisation was taken into account compared to larger network sizes in which values stayed roughly the same. Still, the random hierarchy obtained the highest levels of hierarchy complexity in both indices of all the models, indicating that correcting for multi-ordered degrees would not alter previous conclusions of hierarchical complexity for these models[5].

In summary, node heterogeneity was prominent in scale-free networks, neighbourhood organisation was expressed more by small-world networks and hierarchical complexity was a key attribute of random hierarchy models. All models show instances of neighbourhood similarity, but only in small networks (below size 64), of which it was particularly evident in scale-free models.

References

1. Erdös P. & Rényi, A. On random graphs. *Pubilcationes Math. Debrecen* **6**, 290–297 (1959).
2. Dall, J. & Christensen, M. Random geometric graphs. *Phys. Rev. E* **66**, 016121 (2002).
3. Watts D.J. & Strogatz, S. H. Collective dynamics of small-world networks. *Nature* **393**, 440–442 (1998).
4. Barabási, A.-L. & Albert, R. Emergence of Scaling in Random Networks. *Science* **286**, 509 LP – 512 (1999).
5. Smith, K. & Escudero, J. The complex hierarchical topology of EEG functional connectivity. *J. Neurosci. Methods* **276**, 1–12 (2017).
6. Smith, K., Abásolo, D. & Escudero, J. Accounting for the Complex Hierarchical Topology of EEG Phase-based Functional Connectivity in Network Binarisation. *PLOS ONE* **12**, e0186164 (2017).

3 Data Availability

Code for computing the network models and novel indices are available on the Open Science Framework at doi: 10.17605/OSF.IO/W7BK6.
